# Terahertz field control of in-plane orbital order in La_0.5_Sr_1.5_MnO_4_

**DOI:** 10.1038/ncomms9175

**Published:** 2015-09-18

**Authors:** Timothy A Miller, Ravindra W Chhajlany, Luca Tagliacozzo, Bertram Green, Sergey Kovalev, Dharmalingam Prabhakaran, Maciej Lewenstein, Michael Gensch, Simon Wall

**Affiliations:** 1 ICFO-Institut de Ciències Fotòniques, Av. Carl Friedrich Gauss 3, 08860 Castelldefels, Barcelona, Spain; 2 Faculty of Physics, Adam Mickiewicz University, Umultowska 85, 61-614 Poznań, Poland; 3 Helmholtz-Zentrum Dresden Rossendorf, Institute of Radiation Physics, Bautzner Landstraße 400, 01328 Dresden, Germany; 4 Department of Physics, Clarendon Laboratory, University of Oxford, Oxford OX1 3PU, UK; 5 ICREA-Institució Catalana de Recerca i Estudis Avançats, Lluís Company 23, 08010 Barcelona, Spain

## Abstract

In-plane anisotropic ground states are ubiquitous in correlated solids such as pnictides, cuprates and manganites. They can arise from doping Mott insulators and compete with phases such as superconductivity; however, their origins are debated. Strong coupling between lattice, charge, orbital and spin degrees of freedom results in simultaneous ordering of multiple parameters, masking the mechanism that drives the transition. Here we demonstrate that the orbital domains in a manganite can be oriented by the polarization of a pulsed THz light field. Through the application of a Hubbard model, we show that domain control can be achieved by enhancing the local Coulomb interactions, which drive domain reorientation. Our results highlight the key role played by the Coulomb interaction in the control and manipulation of orbital order in the manganites and demonstrate a new way to use THz to understand and manipulate anisotropic phases in a potentially broad range of correlated materials.

## Abstract (web summary)

Numerous correlated materials exhibit an in-plane anisotropic ground state but their origin is unclear. Here the authors control the orientation of orbital domains in a manganite using the polarization of terahertz pulses, which can be explained by field-induced enhancement of the electron interactions.

## Graphical abstract

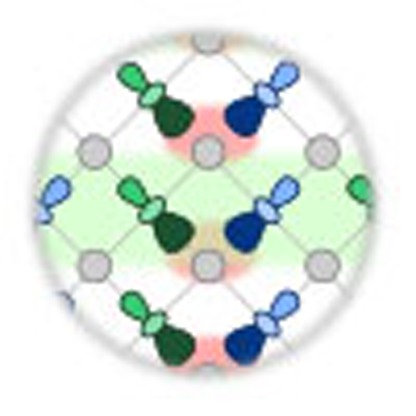

The simultaneous ordering in multiple degrees of freedom is a general phenomenon in layered correlated solids. These ordered states are anisotropic and break the fourfold in-plane symmetry found in the high-temperature phase. In pnictides spin, charge, orbital and structural degrees of freedom lock together at the nematic transition^1^; in the manganites charge, orbital, and structural degrees of freedom simultaneously order to give rise to orbital phases; similar transitions occur in cuprates^2^ and nickelates^3^. Understanding which degree of freedom is primarily responsible for these transitions is a focus of condensed matter physics^1^ and may help to explain why such phases are favoured over alternatives such as superconductivity. Furthermore, dynamic control of the anisotropy, which strongly influences the material properties^4^, could lead to novel devices based on correlated materials.

La_0.5_Sr_1.5_MnO_4_ (LSMO) is a prototypical manganite. The high-temperature state is a paramagnetic semiconductor. The crystal has a layered two dimensional structure of manganese planes as shown in Fig. 1a. Below the orbital ordering temperature, *T*_OO_=230 K, electrons in the twofold degenerate *e*_g_ level of the Mn ions localize on alternating Mn sites. Simultaneously, the occupied *e*_g_ orbitals align. This second-order phase transition breaks the in-plane symmetry, and two possible isoenergetic orbital domains can form. The domains consist of CE-type^5^ zig-zag chains^6^ oriented along one of two possible crystallographic directions, which give rise to anisotropic optical and electronic properties^7^,^8^. Below *T*_N_=110 K, antiferromagnetic order also emerges. The origin of orbital order remains debated, and electronic correlations^9^,^10^,^11^, structural distortions^12^,^13^,^14^,^15^, or spin interactions^16^,^17^,^18^ have each been suggested as the primary factor.

In the following, we address this problem by focusing on the switching of one-domain type to the other under an applied THz field observed using optical birefringence. We show that the multisite Coulomb interaction provides the energy for domain reorientation, even when considering structural distortions. For the single-layer manganite LSMO studied here, field-assisted hopping of electrons from two highly occupied sites onto the same less-occupied site increases Coulomb repulsion and causes domain reorientation when the THz field is orthogonal to the CE chain direction. Our model also suggests that structural distortions create preferential electron hopping directions, which modify Coulomb interactions and may cause domain reorientation in other systems. As Coulomb interactions ultimately drive domain switching regardless of the field-coupling method, our results suggest that electronic corrections are a key factor in the formation of orbital order.

## Results

### Optically probing orbital order

**Figure 1:**
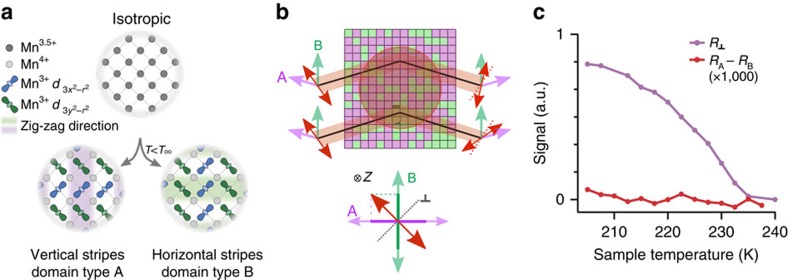
Orbital domain structure in La_0.5_Sr_1.5_MnO_4_. (**a**) Orbital ordering in LSMO. Above *T*_oo_=230 K, the 2D Mn planes are isotropic with a nominal Mn valence of +3.5. On cooling LSMO adopts a CE-type charge and orbital ordering with the two orbital domains shown. The zig-zag stripes show the internal domain anisotropy direction, and the domains are related by a 90° rotation. The orbitals shown are the two *e*_*g*_ orbitals of the Mn ion. (**b**) The optical method used to probe orbital order. (top) Light (red arrow) polarized between the domain directions (purple/green arrows) is rotated left or right on reflection depending on the domain probed. Samples adopt a multidomain structure on cooling and the HeNe probe measures multiple domains. (bottom) Any rotation of the incident light by either domain type will produce a projection in the perpendicular polarization, *R*_⊥_. Measurement of *R*_⊥_ therefore measures the presence of orbital domains without distinguishing between domain types. (**c**) Measure of orbital order and domain structure in LSMO. By measuring the perpendicular component of the reflected light, *R*_⊥_, the orbital order can be measured. Measurements of *R*_A_–*R*_B_ give the net domain alignment, or net optical anisotropy. No *R*_A_–*R*_B_ signal is observed without incident THz light, indicating the sample is in a multidomain state with equal population of both domain types.

We harness the anisotropic reflectance to measure orbital alignment. The polarization of an incident helium-neon laser (HeNe) beam is oriented to lie between the two zig-zag chain directions, A and B (Fig. 1b). Reflected light incident on a domain of Type A experiences a polarization rotation of opposite direction to that experienced by light incident on a domain of Type B. As the HeNe beam is much larger than the orbital domain size^19^, the spatially averaged reflected beam does not experience a net rotation when equal amounts of both domains are present (Fig. 1b). However, both domains have a non-zero projection onto the *R*_⊥_ axis; thus the intensity of *R*_⊥_ as a function of temperature allows us to observe the onset of orbital order, while *R*_A_–*R*_B_ measures the preferential formation of one-domain type (Fig. 1c).

### THz field-induced birefringence

**Figure 2:**
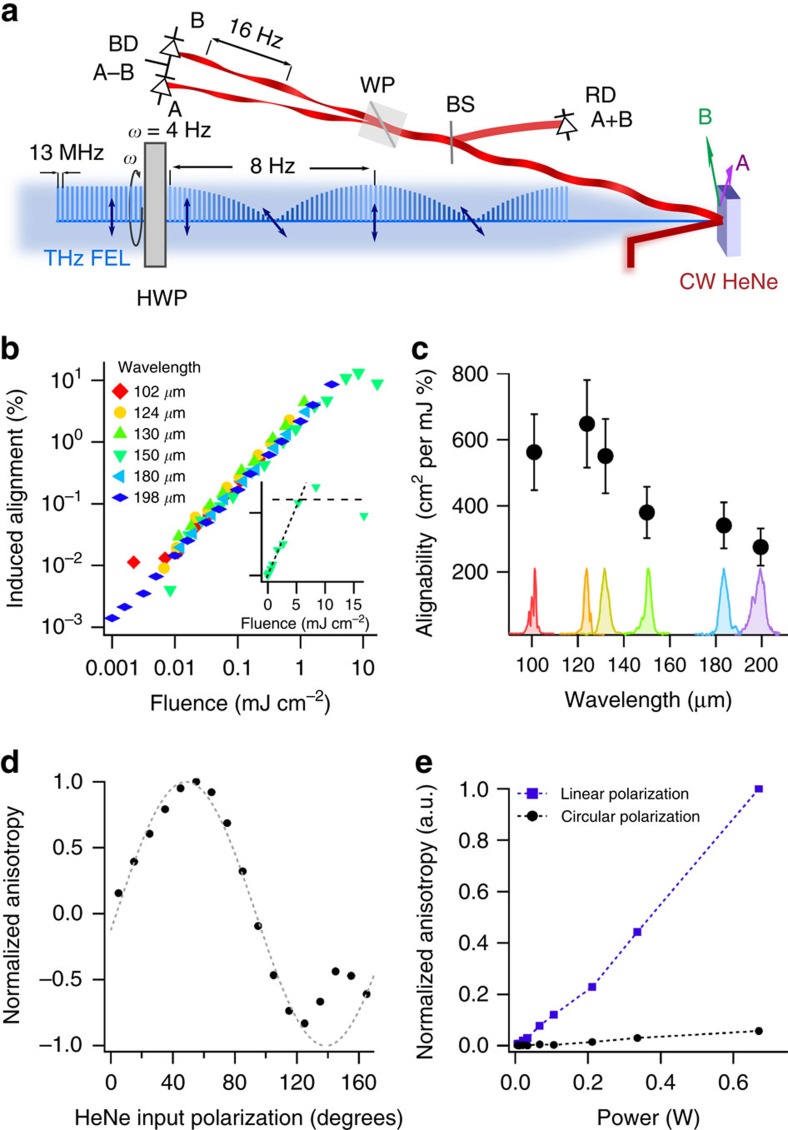
THz-induced alignment of orbital domains. (**a**) 10 ps pulses of linearly polarized THz radiation are generated at 13 MHz from a free-electron laser. The radiation passes through a half-wave plate (HWP) rotating at a frequency *ω*=4 Hz in the plane normal to the beam propagation. This rotates the THz polarization at 2*ω*. The THz is focused onto the sample, which is mounted in a cryostat, through a c-cut quartz window at normal incidence. A continuous wave (CW) HeNe laser arrives collinearly with the THz. Some of the reflected beam is separated by a beam splitter (BS) and recorded by a reference diode (RD) to normalize for changes in total reflectivity from the sample. The remaining reflected HeNe light is split by a Wollaston prism (WP) and collected on a balanced detector (BD) to give a signal which is sensitive to the net optical anisotropy (*R*_A_–*R*_B_). Changes in the anisotropy are detected with a lock-in amplifier at twice the frequency of the THz polarization rotation. (**b**) A log–log plot shows that the power dependence of the anisotropy change is roughly linear before saturating at ∼10%. The insert shows the same data on a linear plot for 150-μm THz light. The data is for a sample temperature of 170 K. (**c**) Induced anisotropy per unit fluence for each of the probe wavelengths. The spectra of the incident THz light are also shown. Error bars show one s.d. (**d**) The dependence of the measured THz-induced anisotropy as a function of probe polarization showing the expected angular dependence. (**e**) THz polarization dependence of the induced anisotropy. The change in anisotropy with circularly polarized THz is substantially reduced and the remaining signal results from a small residual linearly polarized component of the THz field.

Figure 2a shows the experimental set-up for detecting THz-induced domain alignment. Ten-ps-long THz pulses are generated at 13 MHz by a novel infrared free-electron laser (FEL)^20^. The polarization of the THz field is controlled by a mechanically rotating half-wave plate so that the polarization periodically lies along either domain direction A or B. Variations in the polarization rotation of a HeNe probe beam are detected at the rotation rate of the THz polarization using a lock-in amplifier. This enables the measurement of small changes in the probe polarization whilst maintaining a constant isotropic heat load on the sample (see Methods for details). We verified that the measured signal did not change when the rotation rate of the waveplate was changed from 1 to 4 Hz.

Figure 2b shows the THz-induced anisotropy as a function of THz fluence for a sample temperature of 170 K, below *T*_OO_ but above *T*_N_. The THz-induced anisotropy signal was observed to grow linearly with the field intensity for several different excitation wavelengths, and saturation occurred at the highest intensities. By using the literature values for the anisotropy of a single domain^8^, we calculate that we could align ∼10% of the domains before saturation and heating effects dominate. Figure 2c shows that in this resonance-free region the induced anisotropy was weakly dependent on the THz wavelength. Due to the finite bandwidth of the FEL pulses, longer wavelengths have a longer pulse duration and lower peak fields. Thus, as the ability to align domains decreases slightly for longer wavelengths, we conclude that the peak field is more important for domain alignment than the total energy in the THz pulse, which remains constant.

To verify that the observed anisotropy signal was due to domain alignment by the field polarization, we performed several checks. Figure 2d shows the anisotropic signal as a function of the input HeNe probe polarization angle. As expected, when the probe polarization is rotated by 45° to align with an orbital domain axis the polarization state is not modulated by domain alignment, and a further rotation by 45° reverses the polarity of the signal. Figure 2e shows that a largely reduced signal was observed when an additional quarter-wave plate was inserted into the THz beam before the rotating half-wave plate to produce rotating circularly polarized light. The remaining signal is due to a small residual ellipticity in the polarization. This demonstrates the significance of the THz polarization direction and allows us to exclude pointing variations in the THz beam or thermal effects as the primary source of the anisotropy signal.

**Figure 3:**
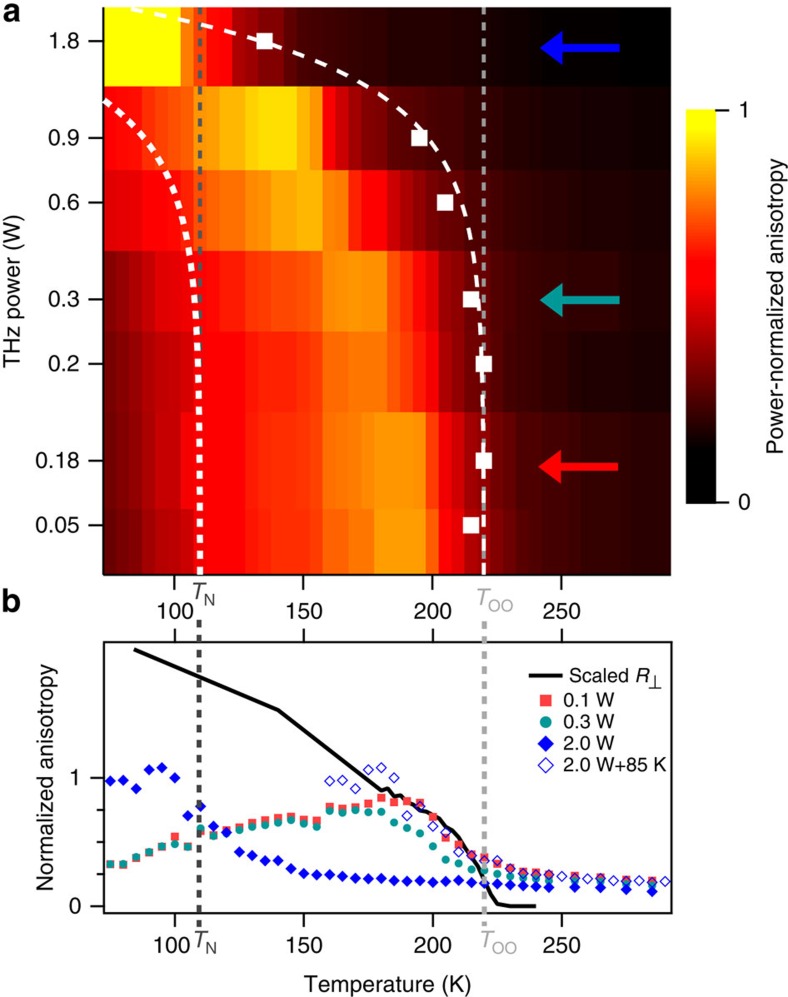
Temperature dependence of THz-induced orbital domain alignment. The 2D plot, (**a**) shows the THz-induced anisotropy signal when normalized by the incident power. Dark dashed lines correspond to the orbital, *T*_OO_, and magnetic, *T*_N_, ordering temperatures. White squares correspond to the onset of the THz-induced signal. THz alignment is only observed for *T*<*T*_OO_. As the temperature approaches *T*_N_ the signal begins to decrease. For higher powers, the onset shifts to lower temperatures due to THz heating of the sample. Dashed white line is a cubic fit to the temperature shift. (**b**) The line-outs for the three indicated powers in **a**. The high-power signal is reproduced with an 85 K offset to demonstrate the heating effect. The solid black line shows *R*_⊥_ from Fig. 1c, indicating how the anisotropic signal changes with temperature.

To understand these observations, we measured a detailed power and temperature dependence. Figure 3a shows the temperature and power dependence of power-normalized THz-induced net anisotropy. On cooling below *T*_OO_, the anisotropic signal increases rapidly for moderate THz powers. This occurs in step with the total orbital order shown in Fig. 1c. This rapid increase is due to the increasing correlation length of domains that occurs as the sample cools. On further cooling, the induced anisotropy saturates at ∼200 K and then starts to diminish, with the signal almost gone below *T*_N_.

For higher THz powers the onset for anisotropy signal is shifted to lower temperatures. This shift can be identified as the result of DC heating, with 2 W of THz power inducing a heating of 85 K. The thermal origin of this shift is clearly seen in Fig. 3b, which shows the temperature dependence of induced anisotropy for different THz powers. At the lowest powers, identical power-normalized temperature dependencies are observed, demonstrating the linear and non-thermal nature of the signal. The high-power signal shows a large thermal shift. If this shift is compensated, the same power-normalized signal is observed. The shift in the rising edge can be used to determine the power dependence of the thermal contribution, which is found to scale as 
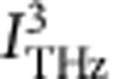
 (white dotted line) and thus only contributes at the highest powers.

### Measured THz field-coupling mechanism

The above results clearly demonstrate that the THz field can be used to align orbital domains, but the question remains as to how this is achieved. Thermodynamically, the energy of a dielectric material in an external field is proportional to *ɛ*(*ω*)*E*^2^ where *ɛ*(*ω*) is the dielectric function at the frequency of the THz field. An anisotropy in the dielectric function at the THz driving frequency would create an energy difference between domains aligned parallel or perpendicular to the THz field. This energy difference is given as Δ*ɛ*(*ω*)*E*^2^, where Δ*ɛ*(*ω*) is the difference between the dielectric function along the different domain directions. Anisotropic dielectric functions can arise as a direct result of the order parameter, as in ferroelectrics, where the polarization modifies the linear dielectric function, or indirectly, as in ferromagnets with magnetostriction, where the magnetic-field-induced strain induces the optical anisotropy.

Although both cases have the potential for field-induced alignment, the switching mechanism will be very different. In the latter case the electric field does not couple to the order parameter directly and instead shifts the thermodynamic potential to favour one-domain orientation. Thermal fluctuations could move the material towards the more favourable orientation. Such a mechanism is unlikely to occur in our experiment as we use pulsed fields which are only ‘on' for ∼10 ps. This makes this indirect process extremely unlikely as the majority of the time the sample is not in an applied field and the two domains are energetically degenerate.

Direct manipulation of the order parameter by THz fields has been achieved through a linear coupling of the field to resonant electric^21^ or magnetic^22^ dipoles. However, to date, domain control with THz fields has not been achieved experimentally. Furthermore, orbital ordering is not described by a macroscopic polarization or magnetization; thus, a linear coupling of the THz field to the order parameter cannot provide the driving force for domain reorientation in LSMO.

### Hubbard model Hamiltonian

To investigate how the THz field can still couple to orbital order, we consider a phenomenological 2D extended Hubbard model for the e_g_ orbitals 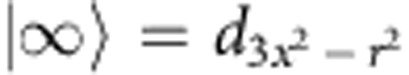 and 
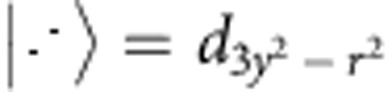
. The Hamiltonian, *H*, can be expressed as


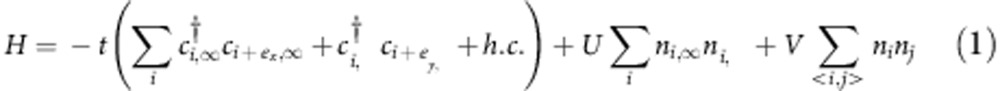


where *U* and *V* are the on-site and nearest neighbour Coulomb interaction terms respectively, *n*_*i*,*α*_ denotes the occupation of the orbital α=∞, and *n*_*i*_=*n*_*i*,∞_+*n*_*i*,_ is the total occupancy of site *i*. The spatial anisotropy of the *d*-orbitals plays a key role in the manganites^6^, and for simplicity we take the limit that electrons can only hop between orbitals of the same character. The hopping between manganese ions decreases the energy of the electrons by *t* and occurs via a bridging oxygen 2*p* orbital. The hopping probability depends on *θ*, the Mn–O–Mn bond angle, as *t*=*t*_0_cos(*π*−*θ*)^2^ where *t*_0_ is the value of the hopping for an undistorted, bond^23^.

**Figure 4:**
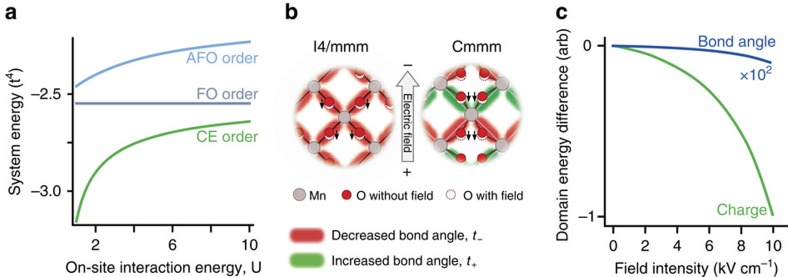
Modelling the coupling between electric fields and orbital domains. (**a**) Calculated energy dependence of three types of orbital order using equation (1): ferro-orbital (FO), where all occupied orbitals are of the same type, antiferro-orbital (AFO), in which the orbital state alternates along both crystallographic directions, and CE order, where the orbital type varies along one axis (antiferro) and is constant along the other (ferro). For a wide range of *U*, the experimentally observed CE order is the lowest energy state, demonstrating that the model can reproduce the correct ground state. Note that the CE phase has two degenerate domains in which the ferro and antiferro directions are rotated. (**b**) Atomic displacements induced by the electric field. Positively charged Mn ions are considered stationary and the relative motion due to the charge disproportionation is neglected. Negatively charged O ions are displaced against the field direction. This displacement changes the Mn–O–Mn bond angle. As the hopping term, *t*, in equation (1) is proportional to the cosine squared of the Mn–O–Mn bond angle, being largest when θ=180°, the hopping decreases for smaller bond angles and increases for larger angles. In the I4/mmm symmetry, the Mn–O–Mn bonds are undistorted (θ=180°), and the bond angle is uniformly decreased. In the Cmmm structure the bonds start distorted, which enables some bond angles to increase in the field, improving the hopping. (**c**) Domain energy splitting when considering the modulation Mn–O–Mn bond angle (blue line) and the field-induced effect. Both models reproduce the experimentally observed scaling with *E*^*2*^ but the field-induced effect stronger by three orders of magnitude.

Figure 4a shows the computed energy diagram for different orbital configurations as a function of the onsite Coulomb energy *U*. As we are studying the state of the system above the magnetic ordering temperature, the spin state is ignored. In this case, with *t*<<*U*,*V*, the experimentally observed CE-type orbital ordering ground state is obtained from a fourth order perturbative expansion of the Hamiltonian, the details of which can be found in Supplementary Note 1 and are summarized in Supplementary Table 1. As expected, the two types of CE-domains are energetically degenerate.

Previous studies on control of magnetic and orbital ordering in LSMO have focused on order melting from optical^24^,^25^,^26^ or vibrationally resonant mid-IR pulses^27^,^28^. The former predominantly influences the charge system, whereas the latter modulates the lattice. Optical excitation triggers charge transfer between Mn sites, directly perturbing the orbital order. Resonant mid-infrared light couples to infrared active phonon displacement that directly modulates the Mn–O–Mn bond angle^29^, which modulates the electronic hopping energy, *t*. We now consider how these two mechanisms can break the degeneracy between the two lowest energy domains obtained from equation (1).

### Competing coupling mechanisms

First, we consider the influence of the field directly on the charge degree of freedom. The THz photon energy is insufficient to trigger charge transfer excitations but instead polarizes the electronic orbitals along the field polarization direction. The effect of the THz field can then be treated quasi-statically in the length gauge as


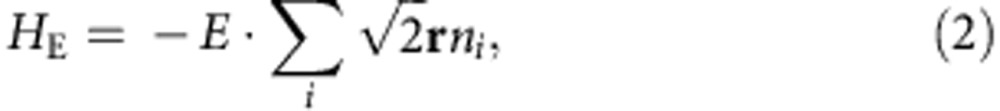


where **r** is the Mn–Mn bond length. We calculate the energy difference of the ground state when the field is applied along or perpendicular to the chain direction and find that the degeneracy of the orbital domains is split. The energy splitting was found to scale as


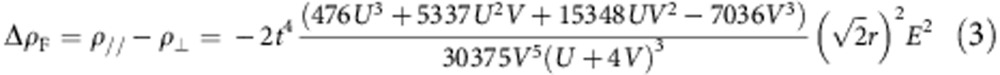


Domains where the field is applied along the chain direction become more stable than those with the field perpendicular to the chains. The model recovers an energy splitting that scales as *E*^*2*^, matching the experimental observation. Details of the calculation are given in Supplementary Note 2.

Second, we consider how the structure distortions induced by the ions in response to the electric field could induce domain alignment. As the oxygen and manganese ions are oppositely charged, they will move in opposing directions in response to the applied field, modifying the Mn–O–Mn bond angle and thus the hopping probability, *t*.

There is some controversy over the crystal structure of LSMO, with some groups reporting the I4/mmm structure above and below *T*_OO_ (ref. 30) and others reporting of a structural transition to a Cmmm phase below *T*_OO_ (ref. 31). In the high symmetry I4/mmm phase the Mn–O–Mn bond angle is *θ*=180°. As shown in Fig. 4b, the result of ionic motion in this phase uniformly decreases all bond angles in the same way. This renormalizes *t* to a lower value, but this does not split the degeneracy of the domain energies. Thus we do not expect to be able to control orbital domains in LSMO through structural distortions induced by the electric field in the I4/mmm phase or in any other material with linear bonds at equilibrium.

However, in the Cmmm space group, the Mn–O–Mn bond angle is decreased to 176.72° at equilibrium. This distortion is, in general, larger in the cubic manganites. In this case, Fig. 4b shows that two bond angles are increased towards the ideal 180° angle and two bond angles are decreased under an applied field. Assuming small changes in bond angle, the change in hopping term becomes *t*_±_= *t*(1±*aE* tan θ), where *aE* is the field-induced change in the bond angle θ, which can be obtained from considering the response of a dipole in an electric field (see Supplementary Note 3). Equation (1) can be re-evaluated with the alternating hopping factors. The resulting domain splitting is given by


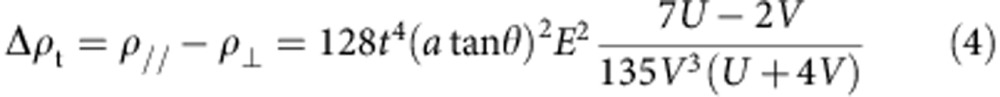


The domain with lower energy is again the domain parallel to direction of the applied field, and the domain energy difference still scales with the intensity of the electric field.

## Discussion

**Figure 5:**
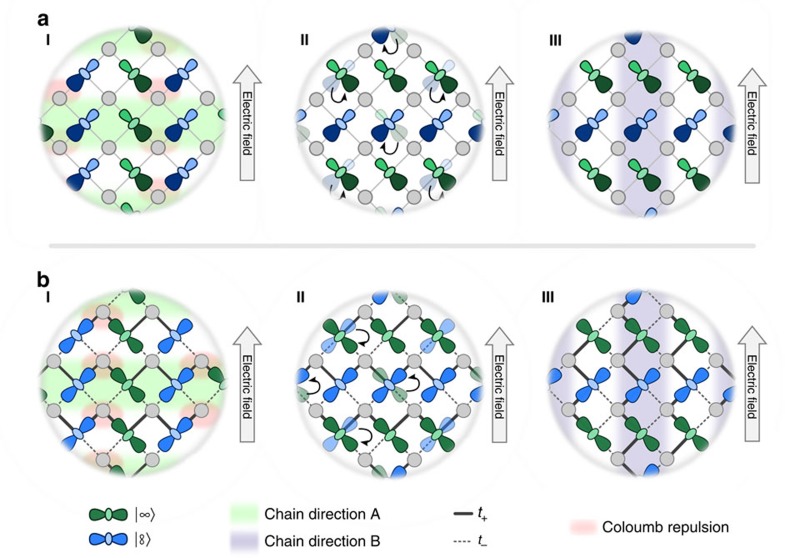
Schematics of the two domain-alignment scenarios. (**a**) The charge driven process. I When the field is applied perpendicular direction of the domain chain, the field causes charge from different Mn^3+^ sites to move towards the same unoccupied Mn^4+^ ion. This has an increased energy cost due to the Coulomb repulsion between the charges. II The energy penalty can cause electrons to change their orbital state so that each electron hops onto a different Mn^4+^ ion. III In the flipped state the orbital domain has been rotated. (**b**) The structural process. **I** When the field is applied along the chain direction, the Mn–O–Mn bond angle is increased and reduced asymmetrically. As a result hopping is increased such that electrons localized on different Mn^3+^ ions are more likely to hop onto the same Mn^4+^ site, again increasing the energy penalty in the field. II Again, the Coulomb energy penalty can be minimized when the orbital rotates and the new orbital pattern (III) is a rotated domain. Note that fields polarized along the crystallographic axis do not induce a domain reorientation for either mechanism.

The domain-switching process that arises from these models are depicted in Fig. 5. In the field-driven case (a), the electric field pushes the charges between the Mn sites. When the field is aligned perpendicular to the domain chain direction, charges from two Mn^3+^ sites are forced on the same Mn^4+^ site. This increases the onsite Coulomb energy penalty and acts as the force to reorientate the domain. If the orbital flips, then the new domain structure is generated. In this case every Mn^3+^ charge is moved onto a separate Mn^4+^ site and thus does not experience the extra Coulomb repulsion.

A very similar process occurs when the domains are aligned due to changes in the tolerance factor (Fig. 5b). Here when the field is aligned perpendicular to the domain direction, the hopping probability is improved such that charge from two Mn^3+^ sites is again moved onto the same Mn^4+^. Like before, the Coulomb energy penalty acts as the force to reorient the domain by minimizing the energy penalty.

By considering the relative strength of the two processes we can determine the most likely driving mechanism in LSMO. Using values of *U*=5 eV, *V*=1 eV and *t*=0.25 eV, based on ref. 32, and the appropriate bond angles for LSMO in the Cmmm phase, we find that 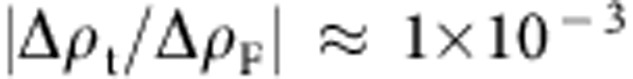. Thus this simple model predicts that field-induced alignment is more likely for LSMO. Structural control is most effective when infrared displacements can induce large anisotropy in the hopping term. This occurs when the system starts in a state with large equilibrium bond angles, enabling larger angular changes for the same oxygen displacement. These displacements may be further enhanced by using fields resonant with the Mn–O–Mn bond. Thus the structural distortion mechanism may dominate in the cubic manganites, such as Pr_0.7_Ca_0.3_MnO_3_, where there are significantly larger initial Mn–O–Mn bond angles.

The model can also qualitatively explain the experimentally observed decrease in domain alignment efficiency when the system magnetically orders. Below *T*_N_ the spins order ferromagnetically along the chains, but antiferromagnetically across the chains^6^. Although perpendicularly oriented domains are still energetically less favourable than parallel ones in the presence of the field, electrons that are transferred across the chains experience an additional energy penalty due to Hund's rule and domains can only switch if the localized 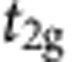 spins also rotates. As the field does not couple to the spin degree of freedom directly, this process only happens due to random thermal fluctuations which are unlikely to occur during the short period of time in which the electric field is applied. Nearest-neighbour spin correlations appear well above *T*_N_ and thus can start to impede domain alignment before long range spin order sets in (ref. 33).

Our results establish that pulsed THz fields can be used to control the anisotropy axis of manganites and represent the first demonstration of THz non-contact control over domains, moving beyond previous studies which examined melting of orbital order by THz fields. Our model shows the importance of Coulomb interactions as a driving mechanism for domain rotation in the manganites and strongly suggests that domain alignment is driven by field-induced hopping in manganites with low structural distortions. Combining the techniques presented here with X-ray based imaging experiments^34^ would further verify the domain-switching process and enable observation of the domain-switching pathway and speed.

Similar experiments should also be applied to other anisotropic phases in the cuprates and pnictides to determine the relative strength of the Coulomb and structural mechanisms in these materials. Furthermore, control of orbital occupation is an emerging method for controlling electronic properties which has typically been achieved with strain or charge transfers from interface states^35^. Our results show that orbital control can be achieved dynamically with THz fields, opening new ways to control device properties and suggest orbital domains could be an alternative to magnetic data storage.

## Methods

### Birefringence measurement

Figure 1c shows the experimental set-up for detecting THz-induced domain alignment. A single crystal of LSMO was grown using the float-zone method, C-cut, optically polished, and mounted in a cryostat with quartz windows. Intense THz light and 632 nm light from a continuous-wave HeNe laser were incident on the LSMO sample at near-normal incidence (<0.5°). The polarization characteristics of the HeNe beam were analysed as a function of THz polarization.

Intense, tuneable, linearly polarized pulses of THz-radiation with Fourier limited pulse durations between 10 and 15 ps are generated at a 13 MHz repetition rate from the FELBE free-electron laser^20^. The THz light was focused to a diffraction limited spot onto the sample using an off axis parabolic mirror with through-hole to permit HeNe co-linearity. The THz polarization was rotated by a mechanical half-wave plate mounted in a motorized rotation stage set to rotate at 4 Hz. This causes a THz polarization rotation at 8 Hz. To check the effect of circularly polarized THz, a fixed quarter-wave plate was placed upstream of the rotating half-wave plate to give rotating circularly polarized THz. This modulation technique keeps the thermal load on the sample constant, while at the same time enabling the detection of the small domain alignment signal. Powers were measured with a calibrated power meter and the fluences reported were calculated by taking the pulse energy, obtained by dividing the power by the repetition rate of the FEL, and dividing by the spot size. THz light was attenuated upstream of the experiment using calibrated attenuators provided by the FEL facility. The THz spot sizes were measured by introducing a flip-mirror between the sample and the focusing off axis parabolic mirror and imaged on a pyroelectric camera (SPIRICON Pyrocam IIIHR).

Linearly polarized HeNe light was used to probe the effect of the THz on the orbital order. The polarization was chosen to lie between the two orbital directions such that there was a non-zero polarization projection into both orbital domains. The HeNe beam was focused to a spot much smaller than that of the THz on the sample, and the reflection was collected. The reflected beam passed through a beamsplitter to provide a reference of the total reflected intensity. The remaining HeNe light passed through a Wollaston prism to separate the light into Type A and Type B components and made incident on a balanced detector. The signal from the balanced detector was fed into a lock-in amplifier triggered at twice the frequency of the THz polarization rotation. This is appropriate as positive and negative THz electric fields produce the same force for domain rotation. The lock-in signal was measured over 10 s of acquisition time, and the mean value was used as the induced anisotropy. To normalize the wavelength dependence of the THz spot size, the induced anisotropy signal was normalized by the wavelength of the THz squared.

## Additional information

**How to cite this article:** Miller, T. A. *et al.* Terahertz field control of in-plane orbital order in La_0.5_Sr_1.5_MnO_4_. *Nat. Commun.* 6:8175 doi: 10.1038/ncomms9175 (2015).

## Supplementary Material

Supplementary Information Supplementary Table 1, Supplementary Notes 1-3 and Supplementary References
