# Supplementary material for: Terahertz field control of in-plane orbital order in La0.5Sr1.5MnO4
Source: Nat Commun. 2015 Sep 18;6:8175. doi: 10.1038/ncomms9175 (PMC4595605; doi:10.1038/ncomms9175)
Supplement: Supplementary Information — Supplementary Table 1, Supplementary Notes 1-3 and Supplementary References [file ncomms9175-s1.pdf]

Supplementary Information

Supplementary Table 1 Effective Orbital Interactions

Effective interactions between orbitals corresponding to different charge configurations on the lattice, as obtained in fourth order perturbation expansion. The corresponding expressions are presented in Eqs.(5-10). The next nearest neighbours in the $\mathbf{x},\mathbf{y}$ directions are collected in the first row, while the diagonal neighbours appear in the lower row.

$(\infty \quad 0 \quad 8), \begin{pmatrix} \infty \\ 0 \\ 8 \end{pmatrix} : A(\mathbf{E})$	$(\infty \quad 0 \quad 8), \begin{pmatrix} \infty \\ 0 \\ 8 \end{pmatrix} : A(-\mathbf{E})$	$(\infty \quad 0 \quad \infty), \begin{pmatrix} 8 \\ 0 \\ 8 \end{pmatrix} : B(\mathbf{E})$	$(8 \quad 0 \quad 8), \begin{pmatrix} \infty \\ 0 \\ \infty \end{pmatrix} : C(\mathbf{E})$
$\begin{pmatrix} 0 & 8 \\ \infty & 0 \end{pmatrix}, \begin{pmatrix} 0 & \infty \\ 8 & 0 \end{pmatrix} : Z^{(1)}(\mathbf{E})$	$\begin{pmatrix} \infty & 0 \\ 0 & 8 \end{pmatrix} : Z^{(2)}(\mathbf{E})$	$\begin{pmatrix} 8 & 0 \\ 0 & \infty \end{pmatrix} : Z^{(2)}(-\mathbf{E})$	$\begin{pmatrix} 0 & \infty \\ \infty & 0 \end{pmatrix} : D(\mathbf{E})$

Supplementary Note 1 Model and perturbation expansion

The complex interplay of spin, charge, orbital and lattice degrees of freedom results in fascinating phenomena in manganite compounds such as *e.g.* colossal magneto-resistance (CMR) and various types of orbital ordering[1]. The CE phase of half-doped manganites with perovskite crystal structure, such as LSMO, is a particularly intricate manifestation of these interactions in manganites, that has attracted much theoretical attention due, in part, to its relevance to the CMR effect[2,3]. In the magnetically ordered phase, it consists of neighbouring ferromagnetically ordered zigzag chains stacked antiferromagnetically. This spin pattern is accompanied by two-sublattice charge ordering and occupation of directed orbitals at occupied Mn^{3+} sites along the zigzag chains. The origin of the CE phase has been the subject of much theoretical debate resulting in various proposals to explain various aspects of this peculiar ordering phenomenon based on: double exchange (kinetic energy gain along the ferromagnetic chains) associated with anisotropy of the hopping integrals of e_g orbitals[4–6], the Coulomb interaction[7–11] and electron-lattice coupling[12–18]including both non-cooperative and cooperative Jahn-Teller effects. However, in many of these cases, magnetic order is either assumed or generated via antiferromagnetic super-exchange interactions, in order to obtain the associated orbital ordering. The residual CE-type insulating, orbital order above the Neel temperature may thus be presumably driven by the competition between kinetic energy, and electronic interactions and/or structural distortions[7,19–21]although a different mechanism based on short-range antiferromagnetic correlations has also been proposed[22].

The transition metal oxides are strongly interacting many body systems that, are in practice, modelled by generalized Hubbard models, incorporating competing interactions between the relevant degrees of freedom[23–25]. In order to obtain a qualitative picture of the energetics behind the THz electric-field induced switching of CE orbital domains, we consider a phenomenological model for the paramagnetic phase of 50% hole-doped manganites. We use a simple, purely electronic, extended Hubbard model with two orbitals per site, describing the active e_g orbitals $|\infty\rangle \sim d_{3x^2-r^2}$

and $|8\rangle = d_{3y^2-r^2}$ directed in the **x** and **y** directions respectively, to reproduce the type of observed orbital domains in a single square layer of the material:

$$\mathrm{H} = -t\left(\sum_i c_{i,\infty}^{\dagger} c_{i+\mathbf{e}_{\mathrm{x}},\infty} + c_{i,8}^{\dagger} c_{i+\mathbf{e}_{\mathrm{y}},8} + h.c.\right) + U\sum_i n_{i,\infty} n_{i,8} + V\sum_{<i,j>}(n_{i,\infty} + n_{i,8})(n_{j,\infty} + n_{j,8}) \quad (1)$$

where U and V are the on-site and nearest neighbour Coulomb interaction terms respectively, with $n_{i,\alpha}$ denoting the electron occupation in the active orbital $\alpha = x, y$ on site i. The spatial anisotropy of the *d*-orbitals plays an important role in the properties of the manganites and results in different hopping terms in the **x** and **y** directions (see e.g. ref 3).

In the Hamiltonian above, we make an approximation and assume only anisotropic hopping between the same orbitals such that electrons in the ***x*** (***y***) orbital hop along the ***x*** (***y***) -direction. The e_g spins are also coupled to localized core t_{2g} spins which can be modelled via the Hund's coupling[3]. In the strong Hund's coupling limit and assuming the t_{2g} spins to be classical, this interaction induces the alignment of the e_g spin with the core spin which can be effectively described to yield a bandwidth narrowing factor. In the paramagnetic phase, the core spin is averaged out leading to a spinless fermion model with effective hopping (including the band narrowing factor) denoted here by t.

The main effect of the THz electric field on the orbitally ordered states can be understood by assuming a stationary field linearly polarized in the plane of the LSMO layer. This is a valid approximation as the THz field frequency is at least an order of magnitude smaller than the hopping dynamics time scales. We describe the electric field $\boldsymbol{E} = E(\boldsymbol{e_x} + \boldsymbol{e_y})$ in the length gauge and consider it, for simplicity, to be polarized at an angle $\pi/4$ with respect to the lattice basis vectors:

$$\mathrm{H_E} = -\boldsymbol{E} \cdot \sum_i \sqrt{2}\boldsymbol{r}\, n_i \quad (2)$$

where n_i is the total electron occupation at site i.

The model Hamiltonians Eqs. 1 and 2 are of the form $H = T + H_0$ where H_0 consists of terms diagonal in the Fermi occupation number operators $n_{i,\alpha}$. In manganite compounds, the hopping amplitude is much smaller than the interaction energy scales $t \ll U, V$ (ref. 3) and is treated as a perturbation below. The subspace of insulating, charge ordered states with one electron per site localized on a sublattice is the unperturbed ground state subspace at 50% hole doping, in the absence of the electric field **E**.

Since the interaction terms do not distinguish between orbital configurations, this subspace is exponentially degenerate. We consider the effect of the hopping T on this subspace both for $\mathrm{E} = 0$ and electric field polarized along the diagonal of the square lattice $\mathrm{E} \neq 0$. These can be treated on the same footing as the electric field term, which is diagonal in the occupation number operators, commmutes with the interaction terms.

The effective Hamiltonian up to fourth order in the perturbation T is given by[26,27]:

$$H_2 = P_0 T \frac{1-P_0}{E_0 - H_0} T P_0 \quad (3)$$

$$H_4 = P_0 T \frac{1-P_0}{E_0 - H_0} T \frac{1-P_0}{E_0 - H_0} T \frac{1-P_0}{E_0 - H_0} T P_0$$

$$-\frac{1}{2}\left(P_0 T \frac{1-P_0}{(E_0-H_0)^2} T P_0 T \frac{1-P_0}{E_0-H_0} T P_0 + P_0 T \frac{1-P_0}{E_0-H_0} T P_0 T \frac{1-P_0}{(E_0-H_0)^2} T P_0\right) \quad (4)$$

where P_0 is the projector on to the mentioned ground state subspace. The second order correction H_2 describing virtual hopping of an electron on to a neigbouring empty site and back does not

distinguish between orbitals occupied. Similarly, the second term in Eq. (4) describes two such sequential processes and also leads to a correction independent of the orbitals involved. Orbital independent corrections do not lift the orbital degeneracy and are henceforth ignored.

Note that since the allowed hopping direction of an electron is determined by the occupied orbital in our model, orbitals cannot exchange places under the action of T. Therefore the first term in H_4 yields only diagonal energy corrections to orbital configurations and one obtains a classical model of interacting orbitals. This perturbative term describes sequential hopping processes through intermediate excited states. The first hopping process always results in a transfer to an excited state with the charge gap $3V \pm E$ where $\pm$ determines whether the electron hopping is against or along the direction of the field. In the second hopping, an electron may hop, if allowed, onto an already occupied site resulting in an energy contribution U, or the second electron in the considered pair hops to an excited state. At this stage the electric field contribution to the excitation energy may be 0 if the two hopping processes occur in opposite directions with respect to the field or $\pm 2E$ if both hops occur against or with the field. The resultant interaction energies of various configurations are produced above (see Supplementary Table 1).

$$A(E) = -t^4\Big(\frac{1}{(3V-E)(U-2E)(3V-E)}\times 1 + \frac{1}{(3V-E)(5V-2E)(3V-E)}\times 4$$
$$+\frac{1}{(3V-E)5V(3V-E)}+\frac{1}{(3V-E)5V(3V+E)}+\frac{1}{(3V+E)5V(3V+E)}+\frac{1}{(3V+E)5V(3V-E)}$$
$$+\frac{1}{(3V+E)(6V+2E)(3V+E)}\times 4$$
$$+\frac{1}{(3V-E)6V(3V-E)}+\frac{1}{(3V-E)6V(3V+E)}+\frac{1}{(3V+E)6V(3V+E)}+\frac{1}{(3V+E)6V(3V-E)}\Big)$$

$$B(E) = -t^4\Big(\frac{1}{(3V-E)(5V-2E)(3V-E)}\times 4 + \frac{1}{(3V+E)(5V+2E)(3V+E)}\times 4$$
$$+\frac{1}{(3V-E)6V(3V-E)}+\frac{1}{(3V-E)6V(3V+E)}+\frac{1}{(3V+E)6V(3V+E)}+\frac{1}{(3V+E)6V(3V-E)}\Big) \quad (6)$$

$$C(E) = -t^4\Big(\frac{1}{(3V-E)(6V-2E)(3V-E)}\times 4 + \frac{1}{(3V+E)(6V+2E)(3V+E)}\times 4$$
$$+\frac{2}{(3V-E)6V(3V-E)}+\frac{2}{(3V-E)6V(3V+E)}+\frac{2}{(3V+E)6V(3V+E)}+\frac{2}{(3V+E)6V(3V-E)}\Big) \quad (7)$$

$$Z^{(1)}(E) = -t^4\Big(\frac{1}{(3V-E)(4V+U)(3V-E)}+\frac{1}{(3V-E)(6V+U)(3V+E)}$$
$$+\frac{1}{(3V+E)(4V+U)(3V-E)}+\frac{1}{(3V-E)(4V+U)(3V+E)}$$
$$+\frac{1}{(3V+E)(5V-2E)(3V-E)}\times 4+\frac{1}{(3V+E)(5V+2E)(3V-E)}\times 4$$
$$+\frac{1}{(3V-E)6V(3V-E)}+\frac{1}{(3V-E)6V(3V+E)}+\frac{1}{(3V+E)6V(3V+E)}+\frac{1}{(3V+E)6V(3V-E)}\Big) \quad (8)$$

$$Z^{(2)}(E) = -t^4\Big(\frac{1}{(3V-E)(4V+U-2E)(3V-E)}\times 4+\frac{1}{(3V-E)(6V+U)(3V+E)}\times 4 \quad (9)$$

$$+\frac{2}{(3V-E)5V(3V-E)}+\frac{2}{(3V-E)5V(3V+E)}+\frac{2}{(3V+E)5V(3V-E)}+\frac{2}{(3V+E)5V(3V+E)}\Big)$$

$$D(E) = -t^4\Big(\frac{1}{(3V-E)(5V-2E)(3V-E)}\times 4+\frac{1}{(3V-E)(5V+2E)(3V+E)}\times 4$$

$$+\frac{1}{(3V-E)4V(3V-E)}+\frac{1}{(3V-E)4V(3V+E)}+\frac{1}{(3V+E)4V(3V-E)}+\frac{1}{(3V+E)4V(3V+E)}\Big)$$

$$+\frac{1}{(3V-E)6V(3V-E)}+\frac{1}{(3V-E)6V(3V+E)}+\frac{1}{(3V+E)6V(3V-E)}+\frac{1}{(3V+E)6V(3V+E)}\Big) \qquad (10)$$

The checkerboard ordered phase defines a new square lattice rotated by $\pi/4$ radians where, in fourth order perturbation, each site now interacts with its nearest as well as next nearest neighbours with configuration energies given by $D, Z^{(1)}, Z^{(2)}$ and A, B, C respectively. Without the electric field, the orbital degeneracy is lifted as can be seen by comparing energies for orbitally ordered states that are typically in competition in doped manganites: the experimentally observed CE type phase corresponding to the ordering vector $(\frac{\pi}{2},\frac{\pi}{2})$ in the original lattice, the FO "ferro-orbital" state where all occupied orbitals are of the same type and the "anti-ferro-orbital" state where each line of the original lattice is filled with only one type of orbital and neighbouring lines have orthogonal orbitals. The energies per site for these configurations follow from the energies of the 8 bonds around a given occupied site:

$$\varepsilon_{\mathrm{CE}} = (2Z^{(1)}(0) + 2D(0) + 4A(0))/4 \qquad (11)$$

$$\varepsilon_{\mathrm{FO}} = (4D(0) + 2B(0) + 2C(0))/4 \qquad (12)$$

$$\varepsilon_{\mathrm{AFO}} = (4Z^{(1)}(0) + 2B(0) + 2C(0))/4 \qquad (13)$$

For a wide window of values $\frac{U}{V} > 1$, the CE type state is seen to be the lowest energy state (see Fig.4 in the main text, with set energy scale $V > 1$). In fact, the closest competing state is the FO state which becomes favorable above the critical value $U \approx 87.3734\mathrm{V}$. We have also checked, using exact diagonalization of an 8-site cluster, that the CE type phase is indeed the ground state of the model Eq. (1) in the limit $t \ll U, V$ with $U \sim V$.

Supplementary Note 2 Effect of electric field on CE type domains

Having obtained a description of the CE type phase, we turn now to the effect of the electric field on the degenerate domain A and domain B states. The validity of the perturbation expansion above is based on assuming that the electric field is off resonant with the interaction energies so that the ideal checker-board charge ordered space remains energetically well separated from other possible charge ordered states. The energies per site in domains A and B are then

$$\varepsilon_{\mathrm{CE_A}}(E) = \frac{2Z^{(1)}(E)+2D(E)+2A(E)-2A(-E)}{4} \qquad (14)$$

$$\varepsilon_{\mathrm{CE_B}}(E) = (Z^{(2)}(E) + Z^{(2)}(-E) + 2D(E) + 2A(E) - 2A(-E))/4 \qquad (15)$$

The inequivalence of the zigzag chains, i.e. the nearest neighbour interactions in the rotated lattice along the chains, determine the energy level splitting

$$\Delta\varepsilon = \varepsilon_{\mathrm{CE_A}}(E) - \varepsilon_{\mathrm{CE_B}}(E) = (Z^{(2)}(E) + Z^{(2)}(-E) + 2D(E) + 2A(E) - 2A(-E))/4 \qquad (16)$$

For weak fields $E \ll U, V$, this splitting grows quadratically with the electric field, i.e. linearly with the intensity. This is a manifestation of the invariance of the ground state configuration with respect to reversal of the electric field.

$$\Delta\varepsilon \rightarrow -2t^4 \frac{(476U^3+5337U^2V+15348UV^2-7036V^3)}{30375V^5(U+4V)^3} E^2 \tag{17}$$

The domain A configuration, with zigzag chains aligned along the direction of the field thus becomes energetically more favourable than domain B and represents a more stable thermodynamic phase. Therefore, in the experimental situation, wrongly aligned domains should be expected to undergo rotation of orbitals to the energetically favourable alignment.

In the above, we have not taken into account structural distortions and the corresponding electron-lattice interactions, since the THz field couples weakly and non-resonantly to the phonon degrees of freedom (however see section below). Thus, for simplicity, we have also aimed for a description of the field-less phases purely in terms of electronic degrees of freedom using a simplified hopping matrix Eq. (1) which yields the CE-type phases. A more realistic model would include the effects of non-orthogonality of the elongated orbitals $d_{3x(y)^2-r^2}$, and can be most succinctly captured by the hopping matrix in terms of an orthogonal e_g basis given by Slater-Koster integrals (see *e.g.* ref. 3 for a comprehensive review of theoretical approaches to magnetically ordered manganites incorporating the interplay of spin, orbital and charge degrees of freedom). In this case, in the paramagnetic phase, the CE orbital ordered phase with elongated orbitals at the Mn^{3+} sites has, in fact, been shown to be stabilized by anharmonic contributions to the elastic energy associated with structural distortions[7,21]. Thus our approximate approach captures the essential qualitative features of a more realistic model for CE type orbital ordering. Similarly, the effect of the electric field E on the relative stability of the states corresponding to the two kinds of domains will be qualitatively similar in the two models. Indeed, even though the perturbative corrections would include contributions due to the gain in elastic energy, as well as new hopping amplitudes, the electric field energetically favours hopping along the field which will result in a splitting in energies of the two domain states. Furthermore, since the physics is symmetric with respect to the inversion $\mathrm{E} \rightarrow -\mathrm{E}$, the lowest order splitting is $\sim E^2$.

Supplementary Note 3 Electric field induced structural changes

As an alternative to the above purely electronic scenario, we consider the effect of electric field induced static lattice distortion on the two types of CE domain configurations. This lattice distortion can be taken into account primarily as a change of the hopping amplitudes t. Already in the absence of the electric field, the so-called tolerance factor in manganite compounds quantifies a structural distortion which can lead to the deviation of the Mn-O-Mn bond angle from the value π corresponding to ideal cubic symmetry[1,3]. An electric field acting at angle $\pi/4$ with respect to the square lattice basis vectors leads to the dimerization of the hopping Hamiltonian T. Nearest neighbours on the square lattice connected by the same effective amplitude form zigzag chains. Two neighbouring zigzag chains are characterized by different hopping amplitudes t_1 and t_2 (see Fig. 4 in main text). The Hamiltonian thus obtained has the same structure as in Eq. (1) but now with the described dimerized hopping. As in the previous section, fourth order perturbation theory can be invoked to obtain effective interactions between orbitals in the charge ordered subspace. The hopping dimerzation leads to an energy splitting (per site) between the two types of CE type zigzag orbital domains A and B

$$\Delta\varepsilon_{\mathrm{d}} = 2t^4 \frac{(t_1^2-t_2^2)^2(7U-2V)}{270V^3(U+4V)} \tag{18}$$

To model the field dependence on the structure, we assume that only the oxygen ions move relative to the manganese ions which makes a small change in the Mn-O-Mn bond angle. The effective hopping amplitude t can be expressed as $t = t_0 \cos^2(\pi - \theta_0)$, where t_0 is the hopping integral for a flat Mn-O-Mn bond[28]. Small changes in the bond angle, θ_0, change the hopping as $t' = t(1 +$

$2\Delta\theta \tan\theta$) and we neglect effects arising from the movement of the oxygen ion along the Mn-Mn bond direction. When bond angles that determine t_1 and t_2 change in the opposite direction we have $(t_1^2 - t_2^2)^2 = 64(\Delta\theta \tan\theta)^2$.

In order to compare Eq. (18) with Eq. (17) we need to express $\Delta\theta$ as a function of the electric field. For a small displacement Δx of the oxygen ion away from the Mn-Mn bond axis results in a change in bond angle give as $\Delta\theta = 4\Delta x / r_{\mathrm{MM}}$, where r_{MM} is the Mn-Mn distance. The relation between Δx and the electric field can then be obtained by considering the response of a dipole displacement driven by a non-resonant DC field as $qE = -m\omega_0^2 \Delta x$, where q, m, ω_0 are the dipole charge, mass and resonant frequency respectively. This then gives the following,

$$\Delta\theta = \frac{4qE}{\sqrt{2} m \omega_0^2 r_{\mathrm{MM}}} = \alpha E, \quad (19)$$

where the factor of $\sqrt{2}$ comes from the fact that the electric field is at an angle $\pi/4$ to the displacememnt. Equation (18) can then be used to calculate the change in t. In the paper we assume that the dipole charge is approximately the electron charge $q \approx e = 1.6 \times 10^{-19}$C, the dipole mass can be approximated by the oxygen mass $m = m_O = 2.7 \times 10^{-26}$ kg, the restoring force is given by the lowest frequency IR active phonon mode at $\omega_0 = 2\pi \times 10^{-12}$ s^{-1}. Although these quantities are estimates they should be correct to within an order of magnitude. Finally, $r_{\mathrm{MM}} = 3.9 \times 10^{-10}$m and $\theta_0 = 176.72°$ are obtained from diffraction data of LSMO in the C_{mmm} phase[29].

Supplementary References

1. Tokura, Y. *Colossal Magnetoresistive Oxides (Advances in Condensed Matter Science)*. (CRC Press, 2000).

2. Dagotto, E., Hotta, T. & Moreo, A. Colossal Magnetoresistant Materials: the Key Role of Phase Separation. *Phys. Rep.* **344,** 1–153 (2001).

3. Dagotto, E. *Nanoscale Phase Separation and Colossal Magnetoresistance*. (Springer-Verlag Berlin Heidelberg, 2003).

4. Solovyev, I. V. & Terakura, K. Magnetic Spin Origin of the Charge-Ordered Phase in Manganites. *Phys. Rev. Lett.* **83,** 2825–2828 (1999).

5. Van den Brink, J., Khaliullin, G. & Khomskii, D. Charge and Orbital Order in Half-Doped Manganites. *Phys. Rev. Lett.* **83,** 5118–5121 (1999).

6. Solovyev, I. V. Ferromagnetic zigzag chains and properties of the charge-ordered perovskite manganites. *Phys. Rev. B* **63,** 174406 (2001).

7. Mutou, T. & Kontani, H. Charge-Orbital Stripe Structure in $La_{1-x}Ca_xMnO_3$ (x=1/2,2/3). *Phys. Rev. Lett.* **83,** 3685–3688 (1999).

8. Jackeli, G., Perkins, N. B. & Plakida, N. M. Charge- and magnetic-ordering in a two-orbital double-exchange model for manganites. *Phys. Rev. B* **62,** 372–378 (2000).

9. Mishra, S. K., Pandit, R. & Satpathy, S. Charge ordering via electron-electron interactions in the colossal-magnetoresistive manganites. *Phys. Rev. B* **56,** 2316–2319 (1997).

10. Shu, Z., Dong, J. & Xing, D. Y. Phase diagram of half-doped manganites. *Phys. Rev. B* **63,** 224409 (2001).

11. Yao, Z.-J., Chen, W.-Q., Gao, J.-H., Jiang, H.-M. & Zhang, F.-C. Theory for charge and orbital density-wave states in manganite La0.5Sr1.5MnO4. *Phys. Rev. B* **87,** 155103 (2013).

12. Mizokawa, T. & Fujimori, A. Spin, charge, and orbital ordering in Mn perovskite oxides studied by model Hartree-Fock calculations. *Phys. Rev. B* **56,** R493–R496 (1997).

13. Popović, Z. & Satpathy, S. Origin of Charge-Orbital Order in the Half-Doped Manganites. *Phys. Rev. Lett.* **88,** 197201 (2002).

14. Yunoki, S., Hotta, T. & Dagotto, E. Ferromagnetic *A*-Type and Charge-Ordered *CE*-Type States in Doped Manganites Using Jahn-Teller Phonons. *Phys. Rev. Lett.* **84,** 3714–3717 (2000).

15. Hotta, T., Malvezzi, A. L. & Dagotto, E. Charge-orbital ordering and phase separation in the two-orbital model for manganites: Roles of Jahn-Teller phononic and Coulombic interactions. *Phys. Rev. B* **62,** 9432–9452 (2000).

16. Hotta, T., Takada, Y., Koizumi, H. & Dagotto, E. Topological Scenario for Stripe Formation in Manganese Oxides. *Phys. Rev. Lett.* **84,** 2477–2480 (2000).

17. Bała, J., Horsch, P. & Mack, F. Manganites at quarter filling: role of Jahn-Teller interactions. *Phys. Rev. B* **69,** 094415 (2004).

18. Sboychakov, A. O., Kugel', K. I., Rakhmanov, A. L. & Khomskii, D. I. Relationship between orbital structure and lattice distortions in Jahn-Teller systems. *Phys. Rev. B* **83,** 205123 (2011).

19. Calderón, M. J., Millis, A. J. & Ahn, K. H. Strain selection of charge and orbital ordering patterns in half-doped manganites. *Phys. Rev. B* **68,** 100401 (2003).

20. Khomskii, D. I. & Kugel', K. I. Elastic interactions and superstructures in manganites and other Jahn-Teller systems. *Phys. Rev. B* **67,** 134401 (2003).

21. Khomskii, D. & van den Brink, J. Anharmonic Effects on Charge and Orbital Order. *Phys. Rev. Lett.* **85,** 3329–3329 (2000).

22. Solovyev, I. V. Charge Ordering due to Magnetic Symmetry Breaking. *Phys. Rev. Lett.* **91,** 177201 (2003).

23. Roth, L. M. Simple Narrow-Band Model of Ferromagnetism Due to Intra-Atomic Exchange. *Phys. Rev.* **149,** 306–308 (1966).

24. V.L. Pokrovskii, G. V. U. Magnetic Configurations Due to the Orbital Angular Momentum. *Zh. Eksp. Teor. Fiz.* **61,** 859–869 (1972).

25. Kugel', K. I. & Khomskii, D. I. Crystal structure and magnetic properties of substances with orbital degeneracy. *Zh. Eksp. Teor. Fiz.* **37,** 1429–1439 (1973).

26. Kato, T. On the Convergence of the Perturbation Method. I. *Prog. Theor. Phys.* **4,** 514–523 (1949).

27. Freericks, J. K. Strong-coupling expansions for the attractive Holstein and Hubbard models. *Phys. Rev. B* **48,** 3881–3891 (1993).

28. Tokura, Y. & Nagaosa, N. Orbital Physics in Transition-Metal Oxides. *Science.* **288,** 462–468 (2000).

29. Herrero-Martín, J., Blasco, J., García, J., Subías, G. & Mazzoli, C. Structural changes at the semiconductor-insulator phase transition in the single-layered perovskite $La_{0.5}Sr_{1.5}MnO_4$. *Phys. Rev. B* **83,** 184101 (2011).
